# The U-Shaped Association Between Serum Uric Acid and Red Blood Cell Distribution Width in Acute Ischemic Stroke

**DOI:** 10.3389/fphys.2021.631369

**Published:** 2021-08-03

**Authors:** Ning Rong, Pei Zhao, Jin Yang, Qing-Lei Fan, Qiang Zhang, Zhi-Gang Han, Jian Cai, De-Sheng Zhu

**Affiliations:** ^1^Department of Neurology, Baoshan Branch, Renji Hospital, School of Medicine, Shanghai Jiaotong University, Shanghai, China; ^2^School of Clinical Medicine, Dali University, Dali, China; ^3^Department of Radiology, Obstetrics and Gynecology Hospital of Fudan University, Shanghai, China; ^4^Department of Neurology, Renji Hospital, School of Medicine, Shanghai Jiaotong University, Shanghai, China

**Keywords:** acute ischemic stroke, red blood cell distribution width, serum uric acid, uric acid, two-piecewise regression analyses

## Abstract

**Objective:** The U-shaped association between serum uric acid (SUA) and the functional outcome has been found in acute ischemic stroke (AIS). However, it is unclear if SUA is associated with red blood cell morphology in AIS. This study aimed to determine the relationship between SUA and red blood cell distribution width (RDW) in patients with AIS.

**Methods:** A cross-sectional study including 438 consecutive patients with AIS was conducted. SUA and RDW, biochemical parameters that reflect the heterogeneity of red blood cell volume, were evaluated on admission. We evaluated the association between SUA and RDW through linear curve fitting analyses and two-piecewise regression analyses.

**Results:** The association between SUA levels and RDW followed a U-shape in all patients. In females, the values of RDW significantly decreased with the increment of SUA (per mg/dl: β, −1.45; 95% CI: −2.15 to −0.75; *p* < 0.001) in patients with SUA <3.86 mg/dl and increased with the increment of SUA (per mg/dl: β, 0.60; 95% CI: 0.22–0.97; *p* = 0.002) in patients with SUA ≥ 3.86 mg/dl. Similar results were observed in males with the turning point of SUA = 4.82 mg/dl. After adjusting for potential confounders, a U-shaped association between SUA and RDW was maintained in females, but no statistical significance was maintained in patients with SUA ≥ 4.82 mg/dl in males (*p* = 0.206).

**Conclusion:** In the sample of patients with AIS, we found a U-shaped relationship between SUA levels and RDW, with the turning point of SUA (3.96 mg/dl in females and 4.82 mg/dl in males) by the threshold effect analysis.

## Introduction

Acute ischemic stroke (AIS) is closely related to cerebral arteriosclerosis and blood flow disorders, including classic risk factors such as hypertension, hyperlipidemia, diabetes, and atrial fibrillation, and other potential risk factors. Serum uric acid (SUA), the final decomposition product of purine nucleotide metabolism, is one of the potential risk factors for AIS in blood circulation (Chamorro et al., 2002). It has dual neurological protective and deleterious effects on AIS. The clinical and animal studies showed that SUA, as an antioxidant, has a protective effect on the ischemic penumbra lesions and reduces the adverse prognosis in AIS (Amaro et al., 2016; Aliena-Valero et al., 2018). A meta-analysis involving 12,739 patients with stroke showed that higher SUA levels were associated with a better outcome of ischemic stroke (Lei et al., 2019). On the contrary, as an oxidant, high SUA is associated with hypertension, atherosclerosis, metabolic diseases, and cerebrovascular stenosis (Patetsios et al., 2001; Masi et al., 2019). Furthermore, poorer prognosis is associated with very low and very high concentrations of SUA in patients with AIS (Mapoure et al., 2018). Although a U-shaped relationship between SUA and functional outcomes was found in AIS, the specific mechanism is still unclear.

The red blood cell distribution width (RDW), a parameter reflecting the volume of heterogeneity of peripheral red blood cell (RBC), was initially used to distinguish between different types of anemia (Karnad and Poskitt, 1985). In recent years, RDW was used as a new predictive marker and an independent risk factor in cardiovascular and cerebrovascular diseases (Turcato et al., 2017; Hong et al., 2019). RDW is also considered a biomarker of an inflammatory response, and the elevated RDW is associated with the increased risk of dysfunction and mortality of AIS (McMahon, 2019; Song et al., 2019). Nevertheless, few studies discuss the role of RDW in the pathogenesis of AIS.

Based on the current evidence, few studies have explored the associations between SUA and RDW in AIS. Considering that SUA has dual neurological protective and deleterious effects, we hypothesized that there is a correlation between SUA and RDW in AIS, and the specific linear curve is still unclear. Therefore, the purpose of this study was to assess the association between SUA levels and RDW in population with AIS by the linear curve fitting analysis and multiple linear regression analysis. To our knowledge, no study has previously examined the association between SUA levels and RDW in the population with AIS.

## Subjects and Methods

### Ethics

This study was performed according to the principles of the Declaration of Helsinki and was approved by the Ethics Committee of Baoshan Branch, Renji Hospital, School of Medicine, Shanghai Jiao Tong University, Shanghai, China. We obtained informed consent from all patients or their immediate family members before sample collection.

### Design

This was a cross-sectional study designed to explore the relationship between SUA and RDW levels in population with AIS. Consecutive patients with AIS were enrolled in this study from the Baoshan Branch of Renji Hospital in China from January 2018 to August 2019, and the patient data were recorded in the Stroke Registry Database of the hospital.

### Study Subjects

Patients were diagnosed with AIS according to the criteria defined by the WHO (Hatano, 1976) and identified using International Classification of Diseases (ICD) diagnostic codes for AIS [*International Classification of Diseases*, 10th Revision (ICD-10) I63]. Inclusion and exclusion of patients were ascertained through the chart review.

The inclusion criteria were as follows: (1) acute onset of ischemic stroke within 48 h, (2) focal signs of cerebral dysfunction persisting after acute onset, (3) confirmation by CT or MRI of the brain within 24 h after admission; follow-up CT or MRI was performed within 14 days of admission or in any case of neurological deterioration, and (4) age ≥40 years.

The exclusion criteria were as follows: (1) intracerebral hemorrhage, (2) transient ischemic attacks, (3) malignancies such as leukemia and multiple myeloma, (4) kidney diseases such as acute and chronic nephritis and renal calculus, (5) heart failure, anemia, erythrocytosis, and hepatic disease, (5) use of uric acid lowering drugs, (6) acute myocardial infarction, and (7) non-availability of data, including unintegrated clinical and laboratory data, for review.

### Clinical and Laboratory Data

The demographic data, medical history [hypertension, diabetes, coronary heart disease (CHD), and atrial fibrillation], and medication used before admission (antihypertensive drugs, antidiabetic drugs, lipid-lowering drugs, anticoagulant drugs, and antiplatelet drugs) were collected on admission *via* in-person interviews with the patients or their family members.

Fasting venous blood samples were collected on admission and before drug administration, such as intravenous tissue plasminogen activator or any intraarterial revascularization procedure in the emergency room. The SUA levels were measured with commercially available quantitative oxidase method kits obtained from Kehua Bioengineering Co., Ltd. (Shanghai, China). The detection values ranged from 0 to 80 mg/dl for SUA. The SUA reference values in our laboratory were <6.0 mg/dl in women and <7.0 mg/dl in men. RDW was measured by the XFA6100 automatic hematology analyzer, and the detection output value is RDW-SD with the normal range between 35.0 and 56.0 fl.

The blood samples were also collected to measure routine blood indicators [RBC count, white blood cell (WBC) count, platelet count, neutrophils count, lymphocyte count, platelet distribution width, and hemoglobin], blood biochemical indicators [levels of glutamic–oxaloacetic transaminase, total bilirubin, albumin, total cholesterol, low-density lipoprotein (LDL), high-density lipoprotein, triglycerides, fasting blood sugar, homocysteine, neuron-specific enolase, creatinine, and erythrocyte sedimentation rate (ESR)], and clotting index (levels of d-dipolymer and prothrombin time). All the above determinations were performed in the laboratory of the hospital by individuals blinded to the clinical data.

### Groups

The patients in this study were grouped according to the following two criteria: (1) In the baseline characteristics analysis, patients were categorized into the T1 (low), T2 (middle), and T3 (high) groups, according to the tertiles of SUA levels stratified by gender. (2) In the two-piecewise regression analysis, groups were organized by threshold values and normal reference values of the SUA level. The normal range of SUA levels was <6 mg/ml in women and 7 mg/ml in men.

### Statistical Analysis

The baseline characteristics are presented according to the tertiles of SUA levels stratified by gender. Categorical variables were presented as counts and percentages and were analyzed by Fisher's exact test or chi-square test. Continuous variables were reported as the means and SD for data of normal distribution, which were analyzed by the one-way ANOVA analysis, and they were reported as medians and interquartile ranges for data of abnormal distribution, which were analyzed by the Kruskal–Wallis test. The association between SUA levels and RDW was assessed by the linear curve fitting analysis (generalized additive models) and multiple linear regression analysis. We applied a two-piecewise regression model to examine the threshold effect of the SUA levels on the RDW using a smoothing function. The threshold level (turning point) of SUA was determined by using the threshold effect analysis. Baseline variables that were considered relevant to SUA and RDW or that showed a univariate relationship with RDW were entered into the multivariate linear regression model. Variables for inclusion were carefully chosen, given the number of events available, to ensure parsimony of the final model. Both non-adjusted and multivariate-adjusted models were applied. Statistical analyses were performed using Statistical Package of the Social Sciences Software version 24.0 (SPSS, Chicago, IL, USA) and R (version 3.4), and statistical graphics were generated using GraphPad PRISM 6 (Graph Pad Software Inc., San Diego, CA, USA). The level of significance was set with a two-tailed *p*-value of <0.05.

## Results

### Baseline Characteristics

**Figure 1:**
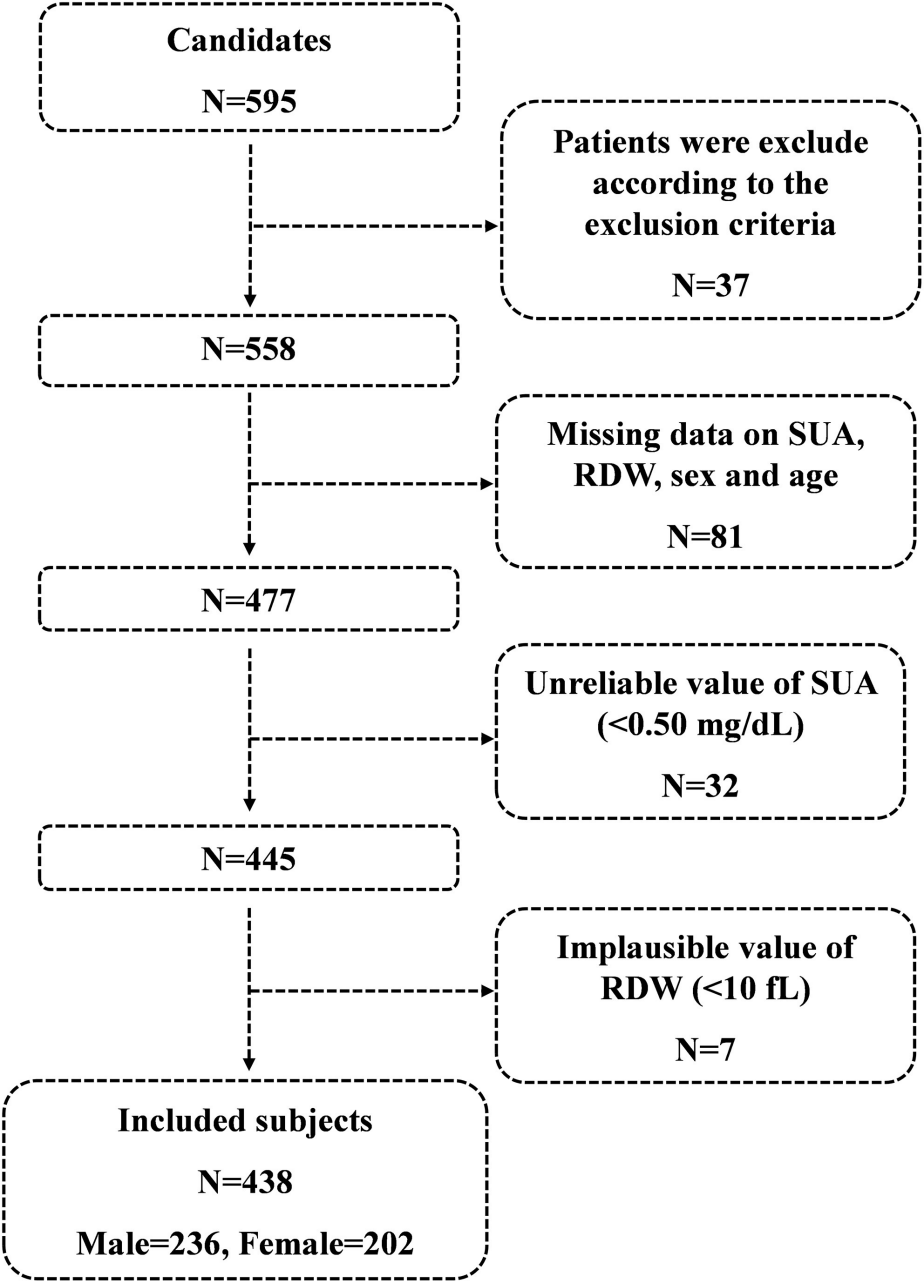
Flowchart of this study.

A total of 595 consecutive candidates were recruited for this study at the time of the final survey in August 2019. Among these candidates, those who had met the exclusion criteria were excluded (*n* = 37) and those who had missing data related to SUA, RDW, sex, and age were excluded from the eligible candidates for this study (*n* = 81). Those with unreliable values of SUA (<0.50 mg/ml) (*n* = 32) and those with implausible values of RDW (<10 fl) (*n* = 7) were also excluded from the pool of eligible candidates for this study. As a result, a total of 438 subjects were included for the final analyses. A flowchart of this study is shown in Figure 1.

Among 438 study subjects, women accounted for 46.12% (*n* = 202) and men accounted for 53.88% (*n* = 236). The age of the enrolled subjects ranged from 40 to 99 years (women, 49–99 years; men, 40–91 years), with a mean age of 73.07 ± 10.83 years (women, 76.47 ± 10.24 years; men, 70.17 ± 10.49 years). The disease duration before admission ranged from 0.5 to 46 h, and the value was abnormally distributed with a median and interquartile range of 7 (4–17.75) h. The SUA ranged from 0.54 to 10.39 mg/ml, and the RDW ranged from 35.20 to 53.50 fl. The mean RDW was significantly the lowest in the T2 group among the tertiles of SUA levels in all patients (*p* < 0.001), and the hierarchical analysis by gender showed significant differences between women (*p* = 0.022) and men (*p* = 0.034). Increasing trends of WBC, neutrophils, LDL, fasting blood sugar, homocysteine, d-dipolymer, and prothrombin time were observed in groups with low (T1) and high (T3) levels of SUA. Higher SUA levels were associated with higher percentages of CHD and AF, decreased levels of platelets and fasting blood sugar, and increased levels of creatinine and ESR at baseline. The baseline characteristics of the included patients are shown in Table 1.

**Table 1:** Baseline characteristics of participants by the tertiles of SUA level.

**Variables**	**Serum uric acid levels, mg/dL**			***p*-value**
	**Tertile 1 (Low)**	**Tertile 2 (Middle)**	**Tertile 3 (High)**	
	**(*N* = 144)**	**(*N* = 147)**	**(*N* = 147)**	
**Basic information**				
Gender (male) (%)	78 (54.17)	79 (53.74)	79 (53.74)	0.996
Age (years)	74.24 ± 10.50	72.39 ± 10.43	72.61 ± 11.50	0.281
Disease duration (hours)	8.00 (5.00–19.12)	7.00 (3.25–18.00)	6.00 (4.00–12.00)	0.157
**Medical history**				
Hypertension (%)	124 (86.11)	130 (88.44)	134 (91.16)	0.399
Diabetes (%)	58 (40.28)	52 (35.37)	43 (29.25)	0.142
CHD (%)	32 (22.22)	35 (23.81)	56 (38.10)	0.004
Atrial fibrillation (%)	1 (0.69)	4 (2.72)	9 (6.12)	0.030
**Blood routine indicators**				
RDW total (fL)	42.51 ± 2.96	41.35 ± 2.36	42.11 ± 2.65	<0.001
RDW Female (fL)	42.62 ± 3.23	41.22 ± 2.54	42.33 ± 3.35	0.022
RDW Male (fL)	42.42 ± 2.73	41.46 ± 2.21	41.92 ± 1.86	0.034
RBC (10^12^/L)	4.35 ± 0.67	4.43 ± 0.57	4.38 ± 0.64	0.528
WBC (10^12^/L)	7.22 ± 2.21	6.84 ± 2.18	7.21 ± 2.66	0.278
Platelets (10^9^/L)	228.56 ± 77.67	223.90 ± 63.53	206.22 ± 62.96	0.014
PDW (fL)	16.14 ± 0.40	16.12 ± 0.36	16.20 ± 0.38	0.120
Neutrophils (10^9^/L)	4.88 ± 2.07	4.42 ± 1.90	4.57 ± 2.02	0.140
Lymphocyte (10^12^/L)	1.60 (1.23–2.31)	1.79 (1.48–2.21)	1.73 (1.42–2.33)	0.326
Hemoglobin (g/L)	127.06 ± 22.31	129.12 ± 19.49	128.13 ± 21.62	0.707
**Blood biochemical indicators**				
SUA (range) (mg/dl)	3.07 ± 0.86 (0.54–4.47)	4.71 ± 0.61 (3.46–5.98)	6.83 ± 1.19 (4.84–10.40)	<0.001
SUA Female (range) (mg/dL)	2.64 ± 0.63 (0.54–3.41)	4.22 ± 0.38 (3.46–4.74)	6.29 ± 1.09 (4.84–8.94)	<0.001
SUA Male (range) (mg/dL)	3.43 ± 0.87 (0.69–4.47)	5.14 ± 0.42 (4.49–5.98)	7.30 ± 1.07 (6.01–10.40)	<0.001
GOT (U/L)	21.24 ± 8.52	20.24 ± 6.83	22.68 ± 5.29	0.156
Total bilirubin (μmol/L)	11.45 (7.90–16.52)	12.10 (9.55–14.95)	11.80 (8.40–16.60)	0.911
Albumin (g/l)	39.76 ± 5.68	40.20 ± 4.94	40.99 ± 4.74	0.118
Total Cholesterol (mmol/L)	4.41 ± 1.07	4.40 ± 1.03	4.60 ± 1.13	0.195
LDL (mmol/L)	2.84 ± 0.98	2.76 ± 0.94	2.92 ± 1.02	0.370
HDL (mmol/L)	1.29 ± 0.38	1.22 ± 0.33	1.25 ± 0.32	0.302
Triglycerides (mmol/L)	1.15 (0.79–1.52)	1.16 (0.88–1.62)	1.17 (0.84–1.54)	0.626
Fasting blood sugar (mmol/L)	6.66 ± 2.83	5.73 ± 1.80	6.09 ± 1.75	0.001
Homocysteine (μmol/L)	14.00 (8.00–25.25)	13.00 (7.00–20.00)	15.00 (9.00–20.00)	0.274
NSE (ng/mL)	13.63 ± 3.16	13.26 ± 3.32	13.81 ± 3.31	0.327
Creatinine (μmol/L)	67.37 ± 32.41	78.43 ± 36.23	94.10 ± 43.14	<0.001
ESR (mm/h)	19.00 (9.75–29.00)	22.00 (11.00–28.00)	24.00 (14.50–32.00)	0.026
D-dipolymer (μg/mL)	0.54 (0.23–1.31)	0.37 (0.22–0.72)	0.39 (0.24–0.67)	0.160
Prothrombin time (second)	11.10 ± 0.90	10.94 ± 0.75	10.96 ± 0.98	0.242
**Medication use before admission**				
Antihypertensive drugs (%)	121 (84.03)	116 (78.91)	128 (87.07)	0.165
Lipid-lowering drugs (%)	56 (38.89)	53 (36.05)	62 (42.18)	0.560
Antidiabetic drugs (%)	56 (38.89)	49 (33.33)	38 (25.85)	0.059
Anticoagulant drugs (%)	0 (0.00)	2 (1.36)	5 (3.40)	0.075
Antiplatelet drugs (%)	124 (86.11)	126 (85.71)	124 (84.35)	0.905

*CHD, coronary heart disease; RDW, red blood cell distribution width; RBC, red blood cell; WBC, white blood cell; PDW, platelet distribution width; SUA, serum uric acid; GOT, glutamic–oxaloacetic transaminase; LDL, low-density lipoprotein; HDL, high-density lipoprotein; NSE, neuron-specific enolase; ESR, erythrocyte sedimentation rate*.

### Association Between SUA Levels and RDW

**Figure 2:**
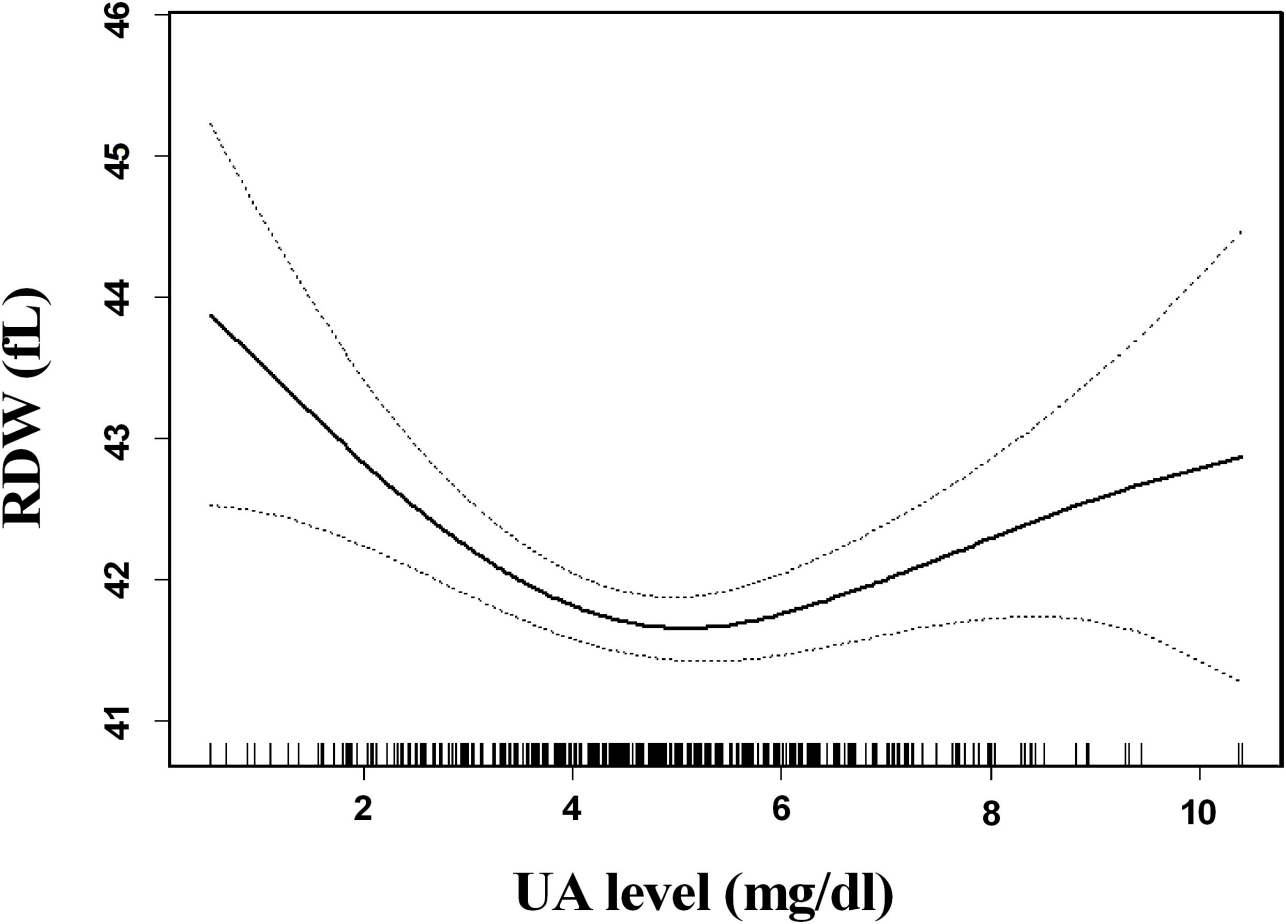
Association between serum uric acid (SUA) levels and red blood cell distribution width (RDW) in all patients. The association between SUA levels and RDW followed a U-shaped relationship in all patients before adjusting for potential confounders (*p* < 0.001). Solid lines represent the fitting curve, and dotted lines represent the corresponding 95% CI.

**Figure 3:**
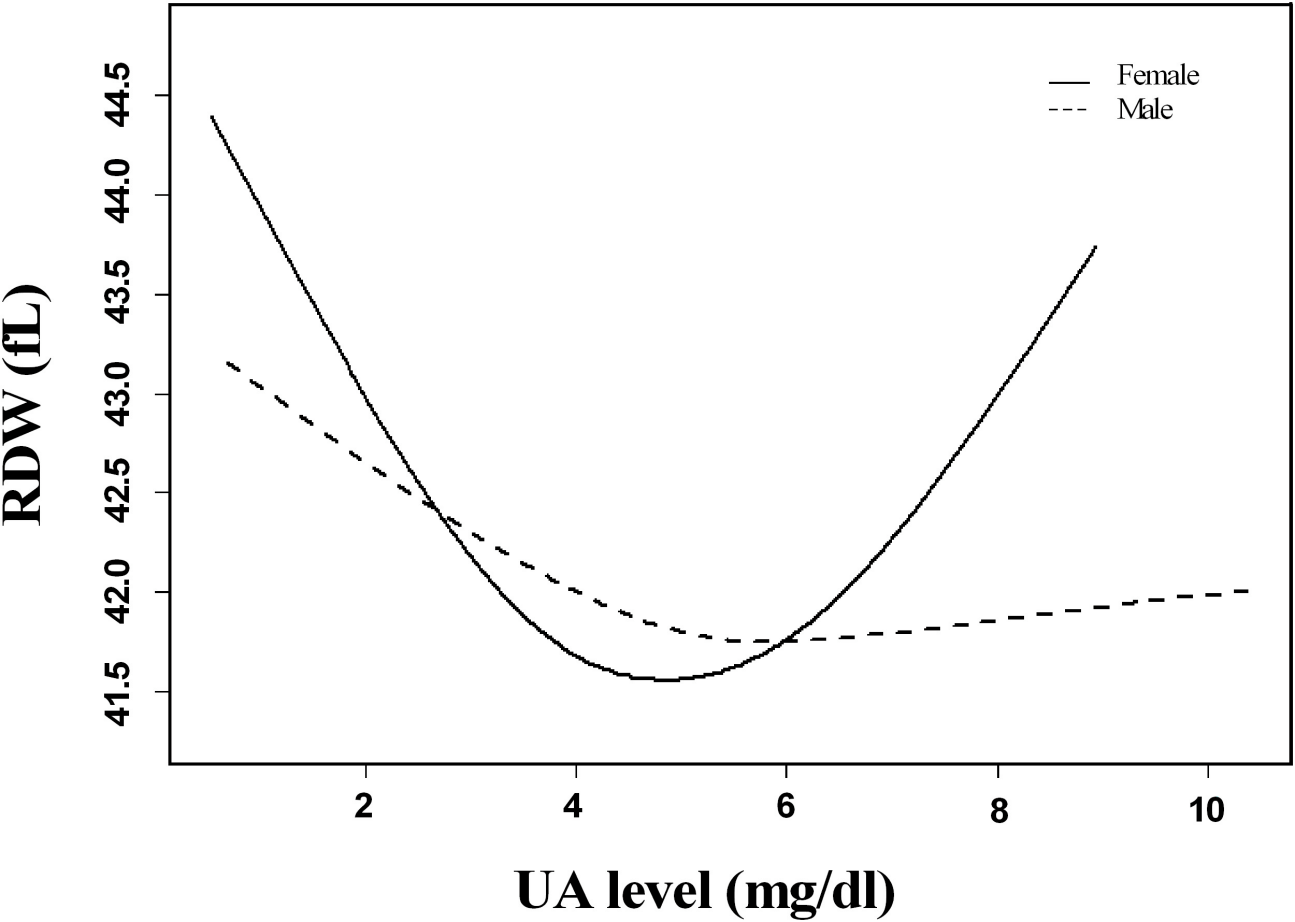
Linear curve fitting of the relationship between SUA levels and RDW by gender. A U-shaped relationship between SUA levels and RDW was detected in women after adjusting for age, hypertension, coronary heart disease, platelets, neutrophils, albumin, fasting blood sugar, creatinine, erythrocyte sedimentation rate, prothrombin time, lipid-lowering drugs, and antiplatelet drugs (*p* = 0.006), but there was no significance of U-shaped relationships in men (*p* = 0.302). Solid lines represent the fitting curve, and dotted lines represent the corresponding 95% CI.

The association between SUA levels and RDW followed a U-shape in all patients before adjusting for potential confounders (*p* < 0.001) (Figure 2). The adjusted smoothed plots suggest that the U-shaped relationship between SUA and the RDW was maintained in females after adjusting for age, hypertension, CHD, platelets, neutrophils, albumin, fasting blood sugar, creatinine, ESR, prothrombin time, lipid-lowering drugs, and antiplatelet drugs (*p* = 0.006), but there was no significance of U-shaped relationship in men (*p* = 0.302) (Figure 3). The univariate analysis of RDW is shown in Supplementary Table 1.

In the threshold effect analysis, in women, the values of RDW significantly decreased with the increment of SUA (per mg/dl: β, −1.45; 95% CI: −2.15 to −0.75; *p* < 0.001) in participants with SUA <3.86 mg/dl and increased with the increment of SUA (per mg/dl: β, 0.60; 95% CI: 0.22–0.97; *p* = 0.002) in participants with SUA ≥ 3.86 mg/dl; in men, the values of RDW significantly decreased with the increment of SUA (per mg/dl: β, −0.84; 95% CI: −1.23 to −0.45; *p* < 0.001) in participants with SUA <4.82 mg/dl and increased with the increment of SUA (per mg/dl: β, 0.29; 95% CI: 0.04–0.53; *p* = 0.026) in participants with SUA ≥ 4.82 mg/dl. After adjusting for age, hypertension, CHD, platelets, neutrophils, albumin, fasting blood sugar, creatinine, ESR, prothrombin time, lipid-lowering drugs, and antiplatelet drugs, the turning point of SUA was 3.96 and 4.82 mg/dl in women and men, respectively, and the U-shaped association between SUA and RDW was maintained in women, but no statistical significance was maintained in patients with SUA ≥ 4.82 mg/dl in men (*p* = 0.206) (Table 2).

**Table 2:** Threshold effect analyses of SUA on RDW using two-piecewise regression models.

**Serum uric acid**	**Model 1 (unadjusted)**		**Serum uric acid**	**Model 2 (adjusted)**	
	***N***	**β (95%CI) *p***		***N***	**β (95%CI) *p***
**Female**	202			202	
<3.86 (mg/dL)	78	−1.45 (−2.15, −0.75) <0.001	<3.96 (mg/dL)	78	−0.89 (−1.51, −0.28) 0.005
≥3.86 (mg/dL)	124	0.60 (0.22, 0.97) 0.002	≥3.96 (mg/dL)	124	0.45 (0.06, 0.84) 0.025
**Male**	236			236	
<4.82 (mg/dL)	99	−0.84 (−1.23, −0.45) <0.001	<4.82 (mg/dL)	99	−0.77 (−1.18, −0.36) <0.001
≥4.82 (mg/dL)	137	0.29 (0.04, 0.53) 0.026	≥4.82 (mg/dL)	137	0.17 (−0.09, 0.44) 0.206

*Model 1: unadjusted*.

*Model 2: adjusted for age, hypertension, CHD, platelets, neutrophils, albumin, fasting blood sugar, creatinine, ESR, prothrombin time, lipid-lowering drugs, and antiplatelet drugs*.

*SUA, serum uric acid; RDW, red blood cell distribution width; CHD, coronary heart disease; ESR, erythrocyte sedimentation rate*.

In addition, when the two-piecewise regression analysis was performed according to normal reference values (females <6 mg/dl, and males <7 mg/dl), it also showed similar results with threshold effect analyses (Supplementary Table 2).

## Discussion

This study identified a U-shaped relationship between SUA levels and RDW in patients with AIS, and the association was significantly maintained in women after adjusting for confounding factors. We further revealed the turning point of SUA (3.96 mg/dl in women and 4.82 mg/dl in men) by the threshold effect analysis. To the best of our knowledge, this is the first study to provide clear evidence of a U-shaped relationship between SUA levels and RDW in patients with AIS.

In this study, we observed an increasing trend of d-dipolymer and prothrombin time in low and high levels of SUA. A previous study has found that both activated partial thrombin time and prothrombin time increased in patients with AIS with high RDW (Rezende et al., 2014). These findings indicate that the blood circulation is in a hypercoagulable state, and the risk of thrombosis increased with the elevated RDW (Ellingsen et al., 2015; Maino et al., 2017). RBC is a component of the thrombus, and the changes in its structure and function can promote the aggregation, degranulation, and recruitment of platelets (Zoller et al., 2014). At the same time, as a determinant of blood viscosity alterations, RBC deformability was correlated with reduced reperfusion in AIS (Allport et al., 2005). In 2012, Kim et al. (2012) found that the elevated RDW either independently or cooperatively affects the blood flow, leads to thrombus, and blocks the blood vessels.

This study showed that the increase of inflammatory indicators such as WBC and neutrophils and the decrease of RBC count in low and high levels of SUA are accompanied by the elevated trend of RDW. Patients without infection and other immune-inflammatory diseases were included in this study. Therefore, the elevated RDW may be related with the potential inflammatory state. In 2015, Weber et al. reported that SUA can stimulate the synthesis of monocyte chemoattractant protein-1, interleukin (IL)-6, and tumor necrosis factor-alpha (TNF-α) and result in the inflammatory response (Weber and Hristov, 2015). The inflammatory state would destroy the maturity of RBCs and elevate the RDW level in peripheral blood (Fujita et al., 2013).

Additionally, this study revealed that the increment of LDL is accompanied by RDW in low and high levels of SUA. A previous study demonstrated that both LDL and SUA were independent predictors of cerebrovascular disease (Chiquete et al., 2013). SUA is significantly associated with oxidized LDL, which is crucial in atherosclerosis and oxidative stress response (Gao et al., 2013). Subsequently, oxidative stress response produces excessive oxygen free radicals, causes oxidative damage to nucleic acids and proteins, resulting in the damage of erythrocyte membrane, and further induces an increase in RDW level (Blum, 2009). Besides the above findings of this study, we also found that the U-shaped relationship between SUA levels and RDW is significant in women but not in men. A previous study has shown that the elevated SUA levels were associated with the risk of stroke in both men and women (Zhong et al., 2017), while the administration of SUA-reduced infarct growth was significant only in women (Llull et al., 2015). Clinical studies and basic animal experiments showed that these differences might be attributed to lower antioxidant capacity in women than in men, the different metabolisms of uric acid, and the biological function of sex hormones in different genders (Miller et al., 2007; Guevara et al., 2009; Zoccolella et al., 2012). The precise mechanism underlying the sex differences in the association between SUA and RDW needs to be further studied.

The mechanism of interaction between SUA and RDW results in AIS is still unclear. Literature showed that it might be related to inflammation, oxidative stress, and neuroendocrine activation. (1) Inflammatory response: SUA, used as an inflammatory mediator, could induce the production of inflammatory factors (e.g., WBC, neutrophils, IL-1ra, IL-6, IL-18, and TNF-a) and activate the inflammatory response of vascular endothelial cells (Ruggiero et al., 2006; Kono et al., 2010). The inflammatory state is significantly correlated with immature erythropoiesis because inflammatory cytokines, such as TNF-a, IL-1b, and IL-6, inhibit the process of erythroid differentiation and maturation in bone marrow (Macdougall and Cooper, 2002). Another study also showed that inflammatory cytokines can inhibit the maturation of erythropoietin-induced erythrocytes *via* inhibiting the bone marrow, resulting in the uneven volume of RBCs and the increase of RDW (Pierce and Larson, 2005). In this study, the levels of inflammatory markers, such as leukocyte and neutrophils, were consistent with that of SUA and RDW. (2) Oxidative stress: A higher level of SUA is associated with stronger oxidative stress, and the latter is positively correlated with the elevated RDW (Semba et al., 2010). Oxidative stress can increase the RDW by impairing iron metabolism and membrane permeability of RBCs, narrowing down the life span of RBCs to modulate the response to erythropoietin through the bone marrow (Pierce and Larson, 2005; Grau et al., 2018). Meanwhile, elevated oxidative stress induces RBCs and endothelial cell adherence and reduces RBCs deformability, leading to poor cerebral infusion (Kaul et al., 2008; Patel et al., 2013). In this study, we observed that the trends in total cholesterol, LDL, and homocysteine were the same as RDW, and these variables were involved in oxidative stress. (3) Neuroendocrine activation: SUA, as an inflammatory medium, has inflammatory reaction leading to the damage of the vascular wall and activates the renin–angiotensin system, and angiotensin II stimulates the generation of erythropoietin, which influences iron metabolism and bone marrow further to increase immature red blood cells in the peripheral blood (Schmidt, 2015; Mateus et al., 2017). Therefore, the interaction between SUA and RDW may be caused by a single factor or multiple mechanisms.

Several limitations should be considered in the interpretation of our results. First, this was a cross-sectional study, and although we did the multiple linear regression analysis and considered several confounder factors, establishing a causal link or the possibility of reverse causation needs further prospective study with a large sample. Second, this study involved Chinese patients with AIS, the majority of who were elderly patients with hypertension with a mean age of about 73 years. This warrants the investigation in other populations to confirm the generalizability of our results. Despite these limitations, all patients who were included in this study did not have malignancies, kidney diseases, heart failure, anemia, erythrocytosis, and hepatic disease, which may affect the values of SUA and RDW and ensures the reliability of the conclusion.

## Conclusion

This study demonstrates a U-shaped relationship between SUA levels and RDW in patients with AIS. Furthermore, we revealed a turning point of SUA (3.96 mg/dl in women and 4.82 mg/dl in men) by the threshold effect analysis. Large cohort studies are required in the future to establish the causal association between SUA and RDW.

## Data Availability Statement

The original contributions presented in the study are included in the article/Supplementary Material, further inquiries can be directed to the corresponding author/s.

## Ethics Statement

The studies involving human participants were reviewed and approved by ethics committee of Baoshan Branch, Renji Hospital, School of Medicine, Shanghai Jiaotong University, Shanghai, China. Written informed consent for participation was not required for this study in accordance with the national legislation and the institutional requirements.

## Author Contributions

D-SZ and NR analyzed the data and wrote the manuscript. PZ, Q-LF, and QZ performed the data curation. Z-GH and Q-LF revised the manuscript. JC and JY conducted the formal analysis. D-SZ and JC supervised this study. All authors read and approved the final manuscript.

## Conflict of Interest

The authors declare that the research was conducted in the absence of any commercial or financial relationships that could be construed as a potential conflict of interest.

## Publisher's Note

All claims expressed in this article are solely those of the authors and do not necessarily represent those of their affiliated organizations, or those of the publisher, the editors and the reviewers. Any product that may be evaluated in this article, or claim that may be made by its manufacturer, is not guaranteed or endorsed by the publisher.
