# Supplementary material for: The U-Shaped Association Between Serum Uric Acid and Red Blood Cell Distribution Width in Acute Ischemic Stroke
Source: Front Physiol. 2021 Aug 3;12:631369. doi: 10.3389/fphys.2021.631369 (PMC8369338; doi:10.3389/fphys.2021.631369)
Supplement: Supplementary file 1 [file Table_1.docx]

**Supplement Table 1. Univariate analysis of RDW**

| **Variable** | **Female** | | **Male** | |
| --- | --- | --- | --- | --- |
|  | **Statistics** | **β (95%CI) *p*** | **Statistics** | **β (95%CI) *p*** |
| Age (years) | 76.47 ± 10.24 | 0.12 (0.08, 0.16) <0.001 | 70.17 ± 10.49 | 0.06 (0.03, 0.09) <0.001 |
| Platelets (10^9^/L) | 231.97 ± 73.91 | -0.00 (-0.01, 0.00) 0.721 | 208.83 ± 62.39 | -0.00 (-0.01, 0.00) 0.361 |
| Neutrophils (10^9^/L) | 4.59 ± 2.15 | 0.35 (0.15, 0.54) <0.001 | 4.65 ± 1.86 | 0.11 (-0.05, 0.27) 0.167 |
| Albumin (g/l) | 39.85 ± 5.35 | -0.18 (-0.25, -0.10) <0.001 | 40.73 ± 4.94 | -0.07 (-0.13, -0.01) 0.031 |
| Fasting blood sugar (mmol/L) | 6.00 ± 1.98 | -0.01 (-0.23, 0.21) 0.918 | 6.29 ± 2.38 | -0.15 (-0.28, -0.03) 0.015 |
| Creatinine (μmol/L) | 79.72 ± 54.17 | 0.01 (0.00, 0.02) 0.019 | 80.34 ± 18.01 | 0.00 (-0.01, 0.02) 0.827 |
| ESR (mm/h) | 25.19 ± 11.42 | 0.04 (-0.00, 0.07) 0.067 | 18.48 ± 12.19 | 0.04 (0.02, 0.07) <0.001 |
| Prothrombin time (second) | 10.93 ± 0.84 | 1.07 (0.59, 1.56) <0.001 | 11.05 ± 0.91 | 0.38 (0.06, 0.70) 0.021 |
| SUA (%) |  |  |  |  |
| Tertile 1 | 66 (32.67) | 0 | 78 (33.05) | 0 |
| Tertile 2 | 68 (33.66) | -1.40 (-2.43, -0.36) 0.008 | 79 (33.47) | -0.96 (-1.68, -0.24) 0.009 |
| Tertile 3 | 68 (33.66) | -0.29 (-1.33, 0.74) 0.577 | 79 (33.47) | -0.50 (-1.22, 0.22) 0.173 |
| Hypertension (%) |  |  |  |  |
| No | 25 (12.38) | 0 | 40 (16.95) | 0 |
| Yes | 177 (87.62) | 0.57 (-0.73, 1.87) 0.391 | 196 (83.05) | 0.41 (-0.38, 1.20) 0.307 |
| CHD (%) |  |  |  |  |
| No | 125 (61.88) | 0 | 190 (80.51) | 0 |
| Yes | 77 (38.12) | 1.52 (0.66, 2.38) <0.001 | 46 (19.49) | 0.57 (-0.17, 1.32) 0.134 |
| Lipid-lowering drugs (%) |  |  |  |  |
| No | 131 (64.85) | 0 | 136 (57.63) | 0 |
| Yes | 71 (35.15) | -0.22 (-1.12, 0.67) 0.624 | 100 (42.37) | 0.20 (-0.40, 0.80) 0.512 |
| Antiplatelet drugs (%) |  |  |  |  |
| No | 36 (17.82) | 0 | 28 (11.86) | 0 |
| Yes | 166 (82.18) | -1.09 (-2.20, 0.02) 0.055 | 208 (88.14) | -0.49 (-1.41, 0.42) 0.292 |
