# Supplementary material for: The U-Shaped Association Between Serum Uric Acid and Red Blood Cell Distribution Width in Acute Ischemic Stroke
Source: Front Physiol. 2021 Aug 3;12:631369. doi: 10.3389/fphys.2021.631369 (PMC8369338; doi:10.3389/fphys.2021.631369)
Supplement: Supplementary file 2 [file Table_2.docx]

**Supplement Table 2.** Two-piecewise regression analyses of SUA on RDW according to normal reference values

| **Serum uric acid** | **Model 1(unadjusted)** | | **Serum uric acid** | **Model 2(adjusted)** | |
| --- | --- | --- | --- | --- | --- |
|  | **N** | **β (95%CI) *p*** |  | **N** | **β (95%CI) *p*** |
| **Female** | 202 |  |  | 202 |  |
| <6 (mg/dL) | 78 | -0.48 (-0.82, -0.13) 0.008 | <6 (mg/dL) | 78 | -0.32 (-0.64, 0.01) 0.058 |
| ≥6 (mg/dL) | 124 | 1.47 (0.64, 2.29) <0.001 | ≥6 (mg/dL) | 124 | 1.03 (0.25, 1.81) 0.010 |
| **Male** | 236 |  |  | 236 |  |
| <7 (mg/dL) | 99 | -0.30 (-0.52, -0.09) 0.007 | <7 (mg/dL) | 99 | -0.27 (-0.51, -0.03) 0.029 |
| ≥7 (mg/dL) | 137 | 0.63 (0.06, 1.20) 0.030 | ≥7 (mg/dL) | 137 | 0.28 (-0.31, 0.87) 0.357 |

Model 1: unadjusted

Model 2: adjusted for age, hypertension, CHD, platelets, neutrophils, albumin, fasting blood sugar, creatinine, ESR, prothrombin time, lipid-lowering drugs, and antiplatelet drugs.
